# Circulating microRNA 132-3p and 324-3p Profiles in Patients after Acute Aneurysmal Subarachnoid Hemorrhage

**DOI:** 10.1371/journal.pone.0144724

**Published:** 2015-12-16

**Authors:** Xian Wei Su, Anna Ho Yin Chan, Gang Lu, Marie Lin, Johnny Sze, Jing Ye Zhou, Wai Sang Poon, Qiang Liu, Vera Zhi Yuan Zheng, George Kwok Chu Wong

**Affiliations:** 1 Division of Neurosurgery, Department of Surgery, Prince of Wales Hospital, The Chinese University of Hong Kong, HKSAR, China; 2 School of Biomedical Science, The Chinese University of Hong Kong, HKSAR, China; Heinrich-Heine University, GERMANY

## Abstract

**Background:**

Aneurysmal subarachnoid hemorrhage (SAH) is a highly morbid and fatal condition with high rate of cognitive impairment and negative impact in quality of life among survivors. Delayed cerebral infarction (DCI) is one the major factors for these negative outcomes. In this study we compared the circulating microRNA profiles of SAH patients and healthy individuals, and the circulating microRNA profiles of SAH patients with and without DCI.

**Methods:**

Peripheral blood samples on Day 7 after the onset of SAH were subjected to microarray analysis with Affymetrix miRNA 3.0 array and quantitative PCR analysis. SAH patients with (N = 20) and without DCI (N = 20) and Healthy controls (N = 20) were included for analyses.

**Results:**

We demonstrated that 99 miRNAs were found to be dysregulated in the SAH patient group with DCI. 81 miRNAs were upregulated and 18 were downregulated. Findings from KEGG pathway analysis showed that miRNAs and target genes for axon guidance and TGF-beta signaling were involved, implying that the resulted differential miRNA expression pattern reflect the results of SAH instead of etiology of the disease. miR-132-3p and miR-324-3p showed distinctive upregulations in qPCR [miR-132: 9.5 fold (95%CI: 2.3 to 16.7) in DCI group and 3.4 fold (95%CI: 1.0 to 5.8) in Non-DCI group; miR-324: 4924 fold (95%CI: 2620 to 7228) in DCI group and 4545 fold (95%CI: 2408 to 6683) in non-DCI group]. However, there were no significant differences in fold changes between SAH patients with and without DCI [fold change ratios (mean+/-SD): 2.7+/-4.2 and 1.1+/-1.1 for miRNA-132 and miRNA-324].

**Conclusion:**

Our study demonstrated that as compared to healthy control, miR-132 and miR-324 showed a upregulation in both SAH DCI and Non-DCI groups. However, the differences between the SAH DCI and non-DCI groups were not statistically significant.

## Introduction

Aneurysmal subarachnoid hemorrhage (SAH) is a highly morbid and fatal condition with high rate of cognitive impairment and negative impact in quality of life among survivors [1] [2]. Rupture of intracranial aneurysms accounts for 85% of all spontaneous subarachnoid hemorrhage and has worldwide and local incidences of 7–10 per 100000 annually [3] [4].

A disproportional high negative outcome impact of SAH is seen especially among patients in the working population [4] [5]. However, the pathogenesis for brain injury after SAH remains complex and incompletely understood [6].

Delayed cerebral infarction (DCI) is a common complication of SAH, which occurs several days after SAH [7]. Factors such as age, initial neurological deficit, aneurysm size, and DCI are important determinant of clinical outcomes [7] [8] [9]. Mechanism of DCI is still not completely understood [10].

In the recent decade, genetic profiling has shifted from DNA level [11],[12],[13],[14],[15] to RNA level after the discovery of microRNAs (miRNAs) and their significance in posttranscriptional gene expression control. miRNAs are non-coding small RNA molecules with a size of 19–25 nucleotides. These small non-coding RNAs bind to mRNAs to regulate both gene and protein expressions by mRNA degradation, negative feedback of gene transcription and inhibition of gene translation [16]. Dysregulation of miRNA levels has been proven to have associations with various diseases such as cancer, cardiovascular and neurological conditions [6], [16]. miRNAs are found to be highly stable in peripheral blood due to its association with Argonaute protein and exosomes [16]. Distinctive patterns of circulating miRNAs have been specifically discovered for vascular diseases such as myocardial infarction, atherosclerotic disease and hypertension [17–19]. For intracranial aneurysms (IA), there were so far four studies up to date related to circulating miRNA profiling [20] [21] [22] [23].

In this study, we compared the circulating microRNA profiles of SAH patients and healthy individuals, and the circulating microRNA profiles of SAH patients with and without DCI.

## Methods

### Patient recruitment and sample collection

The study was approved by the Joint NTEC-CUHK (New Territories East Cluster-Chinese University of Hong Kong) Clinical Research Ethics Committee and written informed consents were obtained from all participants or their next-of-kins. SAH patients were recruited from Prince of Wales Hospital, Chinese University of Hong Kong, Hong Kong SAR, PRC, between 2012 and 2013. SAH were diagnosed by computer tomography angiography (CTA). Fifty-eight SAH patients consented for the study. Peripheral blood samples on Day 7 after the onset of SAH were obtained using EDTA tubes with standard procedures. Samples were placed on ice immediately and centrifuged at 1000 g for 15 min at 4°C. Plasma fraction was aliquoted and stored at -80°C until further analysis. Subsequently, twenty SAH patients with DCI and 20 SAH patients without DCI were selected randomly for further analyses. The control (n = 20) was recruited from the family members of SAH patients with the no major medical problem (without smoking history and hypertension). The control had peripheral blood samples taken and processed similarly.

### Clinical variables and outcome assessment

Neurological status on admission was assessed with the Glasgow Coma Scale [24] and World Federation of Neurological Surgeons (WFNS) scale [25]. Delayed Cerebral Infarction (DCI) was defined as new cerebral infarction noted on discharge or week 2–4 computed tomography of brain, that was not present on the admission and 12–24 hour post-clipping / post-coiling computed tomography of brain. Day 7 was selected for blood withdrawal as it was conceived to be the most relevant time point to investigate the pathological process related to delayed cerebral infarction. Functional outcome was assessed by patient interviews with the modified Rankin Scale (mRS) at 3 months after SAH. Poor neurological outcome was defined as mRS score of 3–6 which indicated moderate-to-severe disability or death.

### Experimental design

At stage one, microarray analysis was performed with SAH patients with DCI and healthy controls to elucidate the miRNA expression patterns using Affymetrix Genechip technology (Affymetrix, Santa Clara, CA) for further investigations. At stage two, quantitative PCR analysis was performed with SAH patients with and without DCI and healthy controls to further investigate the statistical significance of selected miRNA expression levels. miRNA levels between DCI group (SAH patients with DCI) and non-DCI (SAH patients without DCI) group were compared (Tables 1 and 2).

**Table 1:** Age and sex of SAH patients and healthy control in initial microarray screening.

	Control	SAH	*P*
	*N* = 20	*N* = 20	
Age range (y)	18–84	34–90	
Age mean ± SD (y)	49.7 ± 16.7	58.8 ± 12.2	**0.025**
Gender (% Female)	65	45	0.341

**Table 2:** Clinical parameters of SAH DCI group and non-DCI group patients.

	Control	DCI group	Non-DCI group	*P*
	*N* = 20	*N* = 20	*N* = 20	
Demographics				
Age, years	49.7 ± 16.7	58.8 ± 12.2	58.7 ± 11.3	
% Female	65 (13)	45 (9)	65 (13)	
Clinical status on admission				
% GCS ≤ 8	-	25 (5)	20 (4)	0.705
Mean GCS	-	12 ± 4	13 ± 4	0.185
CT feature on admission				
% Fisher Grade 3 or 4	-	100 (20)	100 (20)	1.000
Risk factors				
% With hypertension	-	55 (11)	30 (6)	0.200
% Smokers	-	10 (2)	0 (0)	0.487
Outcome				
% Infarction	-	65 (13)	0 (0)	**<0.001**
% hydrocephalus	-	80 (16)	55 (11)	0.176
mRS 3mo(median, IQR)	-	2, 1–5	1, 0–2	**0.034**
mRS 3mo > 2	-	45 (9)	15 (3)	0.082
mRS 3mo ≤ 2	-	55 (11)	85 (17)	

Data are in % (N) or mean ± SD, or median, interquartle range.

GCS, Glasgow Coma Scale; mRS 3mo, 3-month Modified Rankin Scale score.

Fisher Grade 3 and 4, thick subarachnoid hemorrhage with or without intraventricular/intracerebral hemorrhage.

DCI, aneurysmal subarachnoid hemorrhage patients with delayed cerebral infarction.

Non-DCI, aneurysmal subarachnoid hemorrhage patients without delayed cerebral infarction.

### RNA extraction and quantification

Serums were prepared by adding 20% w/v CaCl_2_ (Sigma, St. Louis, MO) into the plasma samples to a ratio of 1:100 followed by clotting overnight and centrifugation. Total RNA was isolated from pooled serum samples using the miRNeasy Serum/Plasma Kit following the manufacturer’s instructions (Qiagen, Valencia, CA). Serum was thawed on ice and centrifuged at 3000 g for 5 min in a room temperature benchtop centrifuge (Eppendorf, Germany). Aliquots of 200 μl of serum per sample was added with 1 ml of a QIAzol mixture (Qiagen, Valencia, CA) containing 2.5 μg/mL of yeast total RNA (Ambion, Foster City, CA) and the spike-In Control C. Elegans cel-miR-39 miRNA mimics (Qiagen, Valencia, CA). Total RNA was eluted by adding 14 μl of RNase-free water and stored at -80°C. The quantity of the extracted RNA was determined by Nanodrop 2000 UV-Vis spectrophotometer (Thermo Scientific, Waltham, MA).

### Microarray miRNA Analysis

For the initial screening of miRNA expression levels among the SAH patients with DCI (n = 20) and the healthy control (n = 20), samples from each group were combined to form two uniform pooled samples (one from each group) before RNA extraction. Total RNA was isolated from the same volume of serum and a fixed volume of the eluted total RNA was used for array hybridization. The Microarray was performed using Affymetrix GeneChip miRNA 3.0 Arrays kit according to the manufacturer (Affymetrix, Santa Clara, CA). Arrays were scanned by the Affymetrix Scanner 3000 7G (Affymetrix, Santa Clara, CA) after washing and staining. The signal values obtained were analyzed with Affymetrix miRNA Expression Console (Affymetrix, Santa Clara, CA) for data normalization with RMA algorithm [26]. The noise signals were filtered with Detection Above the Background (DABG) workflow [27].

### miRNA qRT-PCR analysis

miRCURY LNA^™^ Universal RT cDNA Synthesis Kit (Exiqon, Denmark) was used for quantitative reverse transcription PCR (RT-PCR) assays as described by the manufacturer. 1 μl of extracted RNA was used for a single RT reaction in a total reaction volume of 20 μl. The cDNA obtained was diluted 1:40 for real-time PCR reactions performed using Applied Biosystems Vii7 Sequence Detection System (Applied biosystems, Waltham, MA) in 10 μl PCR mixtures containing ExiLENT SYBR^®^ Green master mix (Exiqon, Denmark) and specific miRNA primers from the manufacturer. All samples were performed in triplicate. The results were analyzed with the Applied Biosystems SDS software (Applied biosystems, Waltham, MA) for Ct values and melting curve analyses. Relative expression levels were calculated via the 2 –^ΔΔCt^ method [28]. U6 snRNA and cel-miR-39 were used as endogenous and exogenous controls [29], [30].

### Statistical analysis

The microarray data were analyzed with Aligent GeneSpring GX 13 software (Aligent Technologies, Santa Clara, CA) and those with a fold change >1.5 were included in further testings. The microarray data was a single pooled sample exploratory analysis, and thus no statistical significance, confidenced interval, or Bonferroni correction was available. All other data analysis was performed with standard statistical software GraphPad Prism, version 5 (GraphPad, La Jolla, CA). Quantitative data from qPCR experiments were expressed as mean ± SEM unless otherwise specified. Differences between the patient and control groups were analyzed with Mann-Whitney U-test or Kruskal-Wallis test. Uniform error distribution analysis was presented as scattered plots in mean ± SD. Receiver operating characteristic (ROC) curves were constructed using raw ΔCt values using the same statistical software. A value of p < 0.05 was regarded as statistically significant. Fisher’s exact test was performed to test dichotomized or categorical independent variables. Mann-Whitney U-test was used for continuous variables without Gaussian distribution.

## Results

### Clinical features

For microarray investigation, age of SAH patient group with DCI (N = 20) 59+/-12 years and 45% was female. Age of healthy control (N = 20) was 50+/-12 years and 65% was female (Table 1). The clinical features of qPCR analysis cohorts were shown in Table 2.

### Differential miRNA expression pattern found in initial microarray screening

In this study, we profiled genome-wide miRNA expression levels of blood samples in SAH patients and control healthy subjects. Out of 1734 miRNA probesets detected with the microarray panel, 205 were filtered with the DABG background check. The rest was regarded as noise background signals. 99 miRNAs were found to show a dysregulation of expression level with more than 1.5-fold. Among these 99 miRNAs, 81 were upregulated and 18 were downregulated in SAH subjects (GEO accession number GSE72340 and S1 File). KEGG (Kyoto Encyclopedia of Genes and Genomes) pathway [31] analysis was performed with bioinformatics tool DIANA-miRPathv.2.0 [32] with target gene prediction. As listed in S2 File, the dysregulated miRNAs in the patient pooled sample were shown to be involved in prion disease, ErbB signaling pathway, axon guidance, TGF-beta signaling pathway, neurotrophin signaling pathway, and dopaminergic synaptic processes.

### A wide miRNA signal distribution was detected among the healthy individuals in qPCR investigation

**Fig 1:**
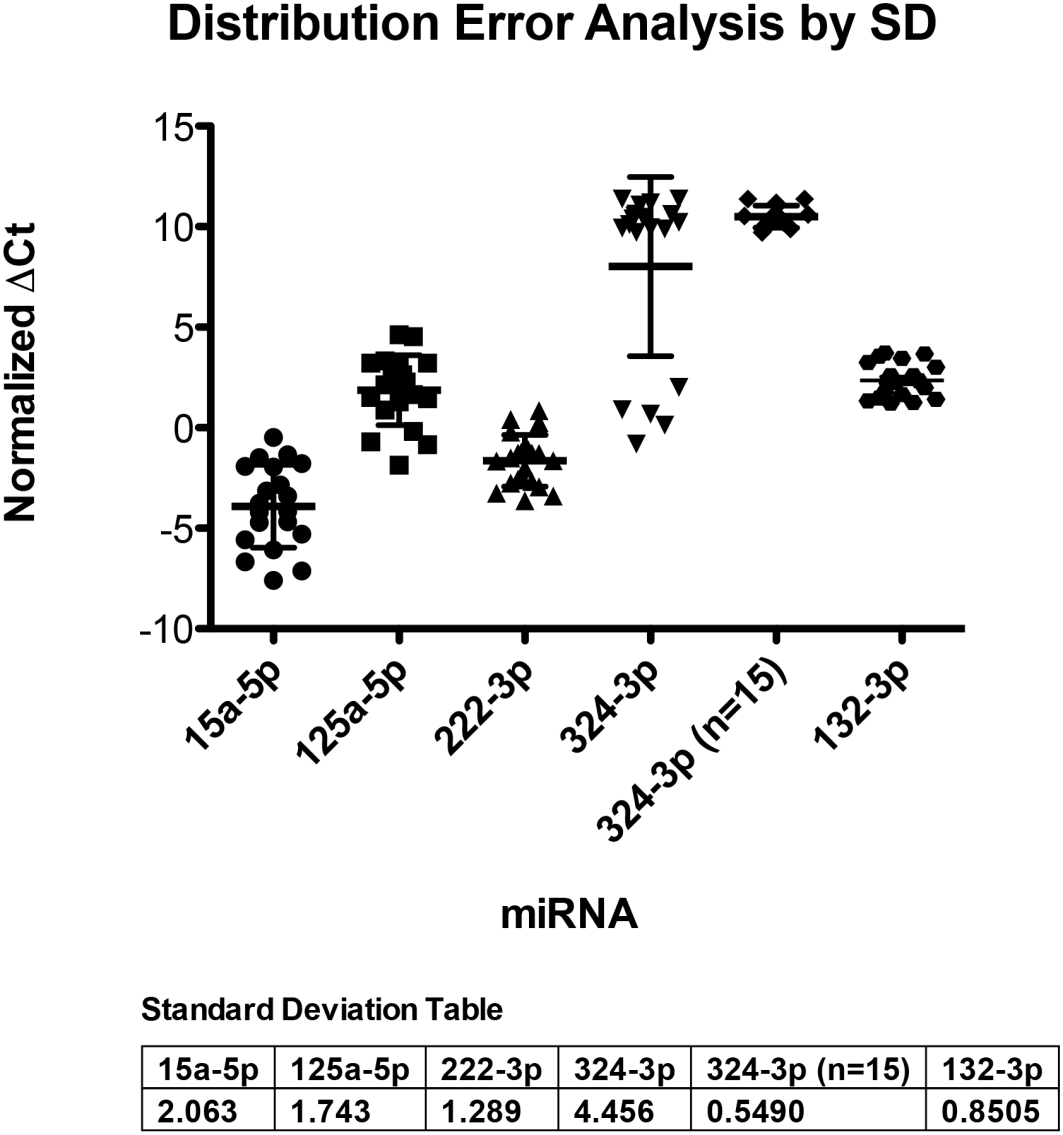
Error Distribution Analysis illustrated by a scattered plot of normalized ΔCt values of the selected five miRNAs in N = 20 healthy control samples (N = 15 undetected in miR-324-3p).

Based on the results observed with the microarray, five miRNAs were selected for further investigation according to their signal intensities and fold change among SAH and control subjects, including miR-15a, miR-125a, miR-132, miR-222 and miR-324. qPCR was performed using designed commercial primers (Exiqon, Denmark) on the N = 20 healthy controls and the expression levels of these five miRNAs were used for error distribution analyzed by their standard deviation. The aim was to determine the suitability of the miRNA to serve as a potential biomarker. A wide signal distribution of detected miRNA expression level in the healthy subjects indicated that a miRNA had a fluctuating baseline. Therefore such miRNAs would be considered as unsuitable for being a potential biomarker. Among the five miRNAs we investigated, miRNA-132 and miRNA-324 (after excluding the five outliers) were selected for further testings (Fig 1).

### Identification of miR-132 and miR-324 as potential biomarkers of SAH

**Fig 2:**
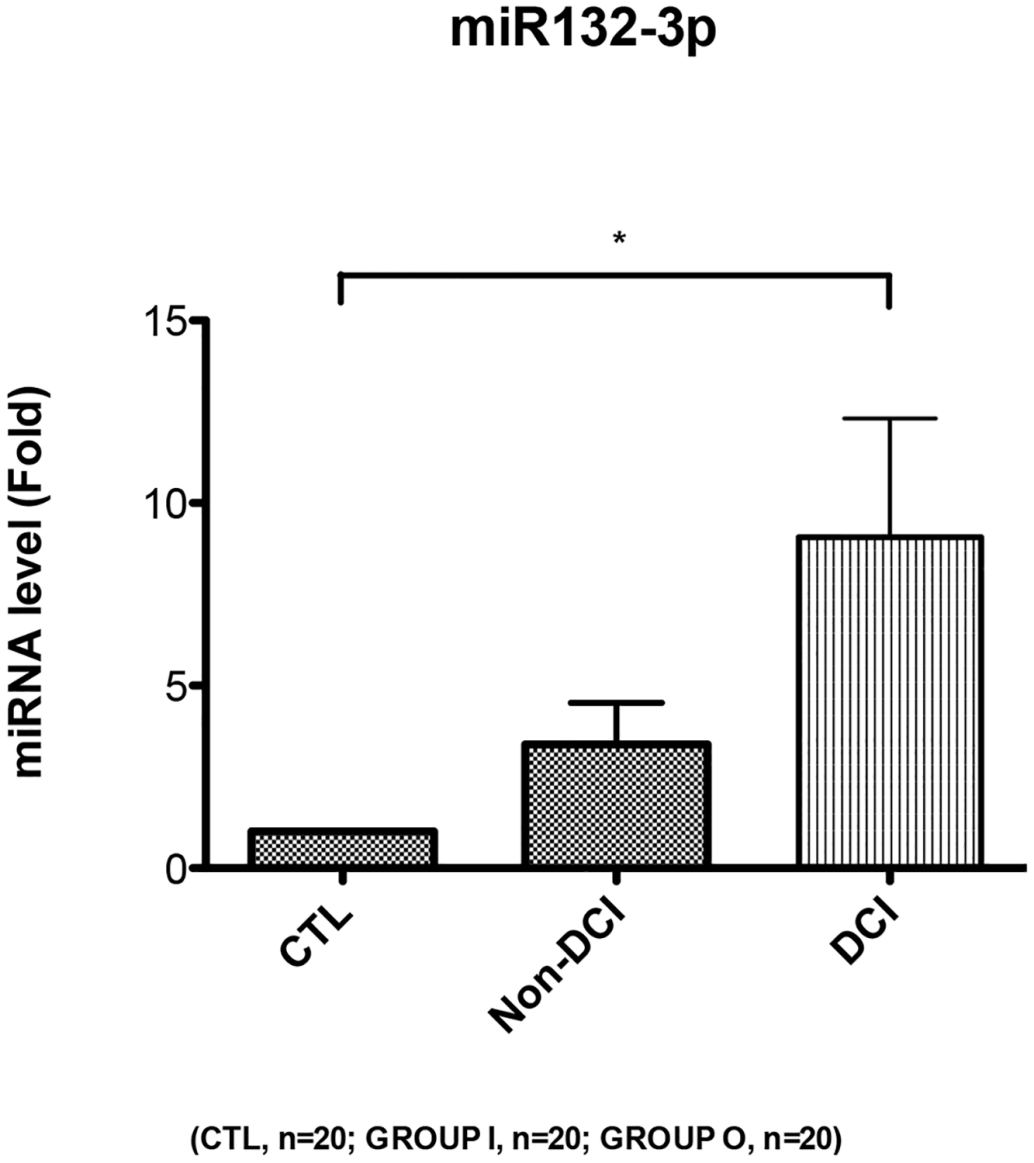
Relative fold change of miRNA expression level in miR-132-3p. *p < 0.05. Data are expressed as mean ± SEM. CTL: Healthy control, non-DCI (Group O): Aneurysmal subarachnoid hemorrhage patients without delayed cerebral infarction, DCI (Group I): Aneurysmal subarachnoid hemorrhage patients with delayed cerebral infarction.

**Fig 3:**
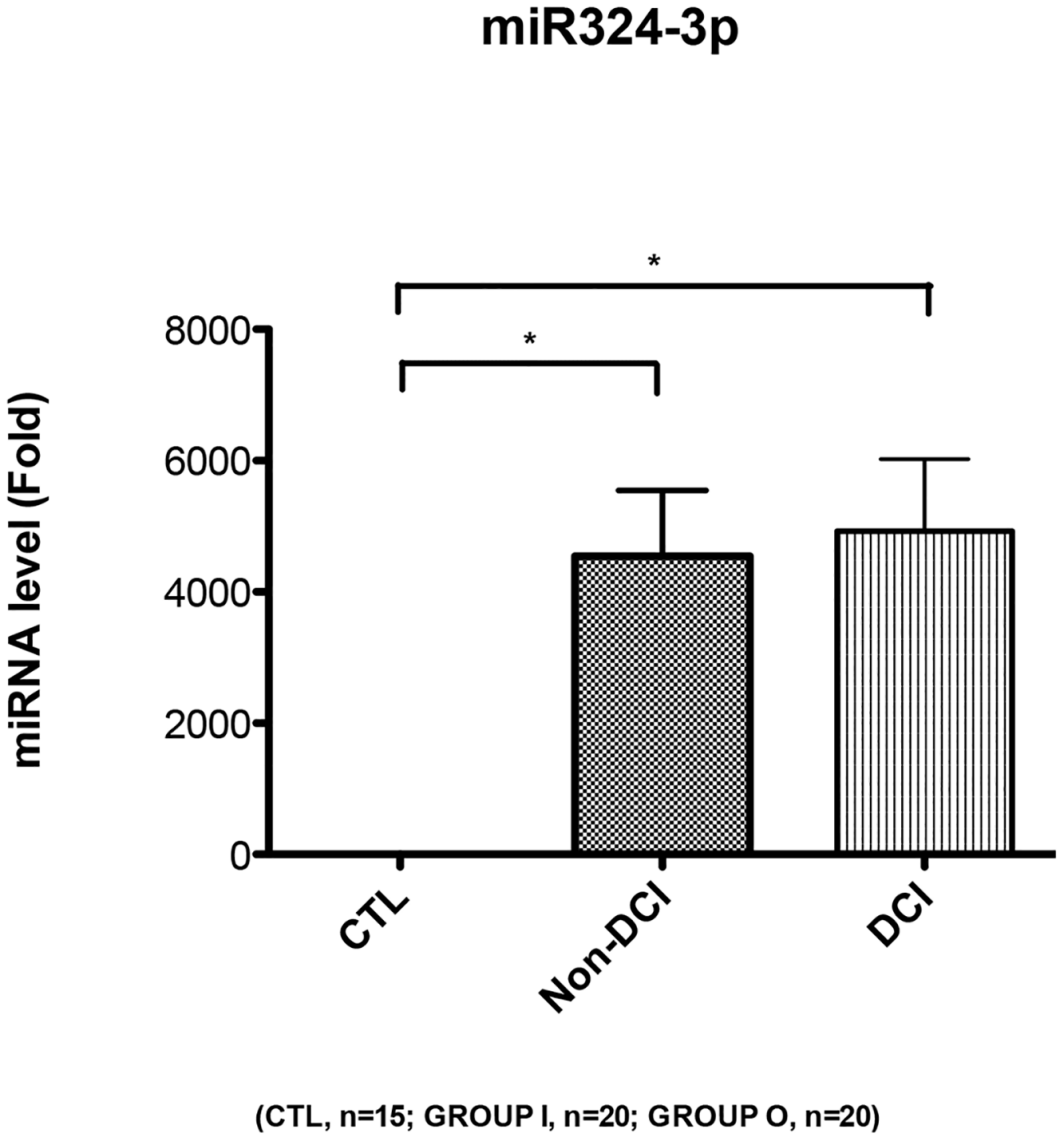
Relative fold change of miRNA expression level in miR-324-3p. *p < 0.05. Data are expressed as mean ± SEM. CTL: Healthy control, non-DCI (Group O): Aneurysmal subarachnoid hemorrhage patients without delayed cerebral infarction, DCI (Group I): Aneurysmal subarachnoid hemorrhage patients with delayed cerebral infarction.

**Fig 4:**
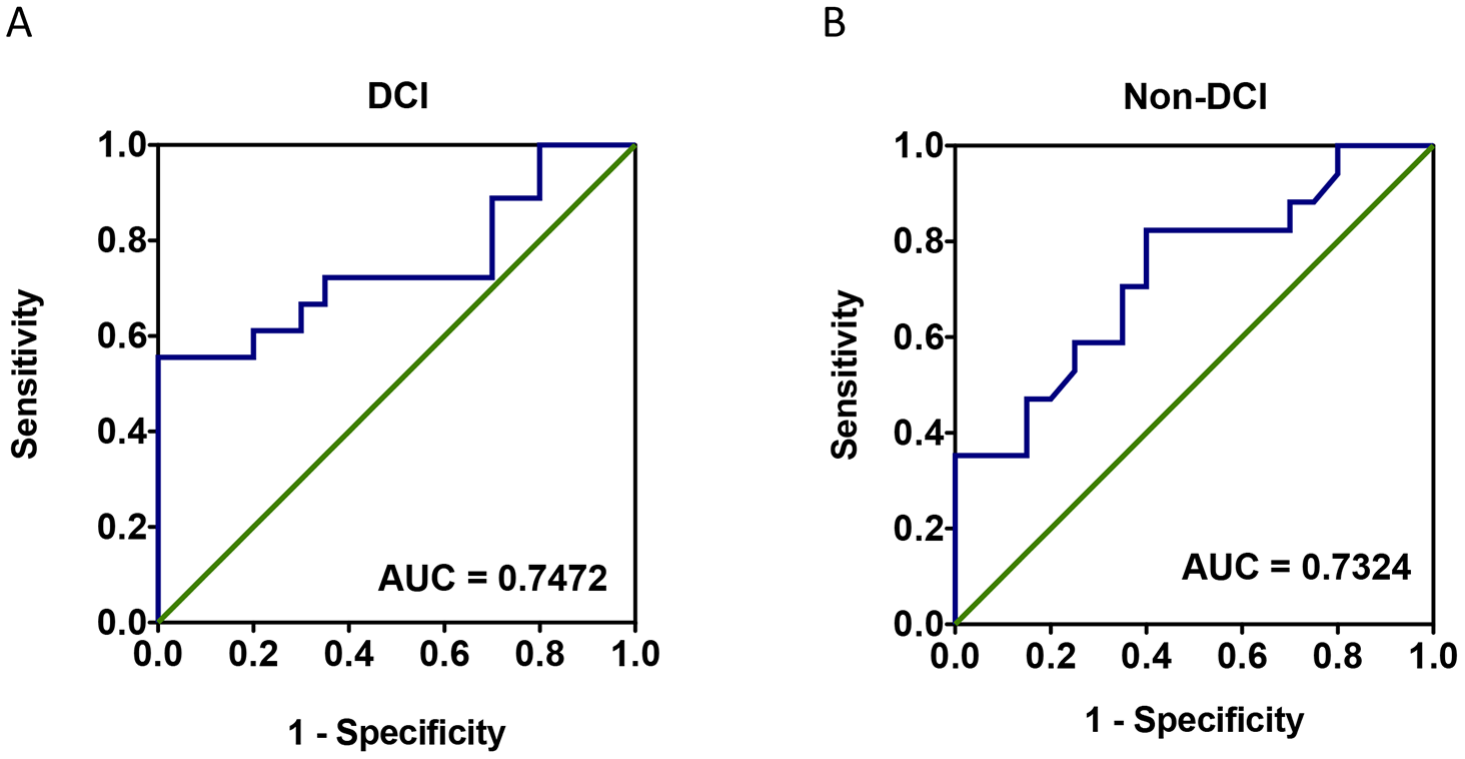
Receiver operating characteristic (ROC) curve of miR-132-3p for aneurysmal subarachnoid hemorrhage patients with delayed cerebral infarction (A) and without delayed cerebral infarction (B) using raw ΔCt values. *p < 0.05. AUC = Area under the curve.

**Fig 5:**
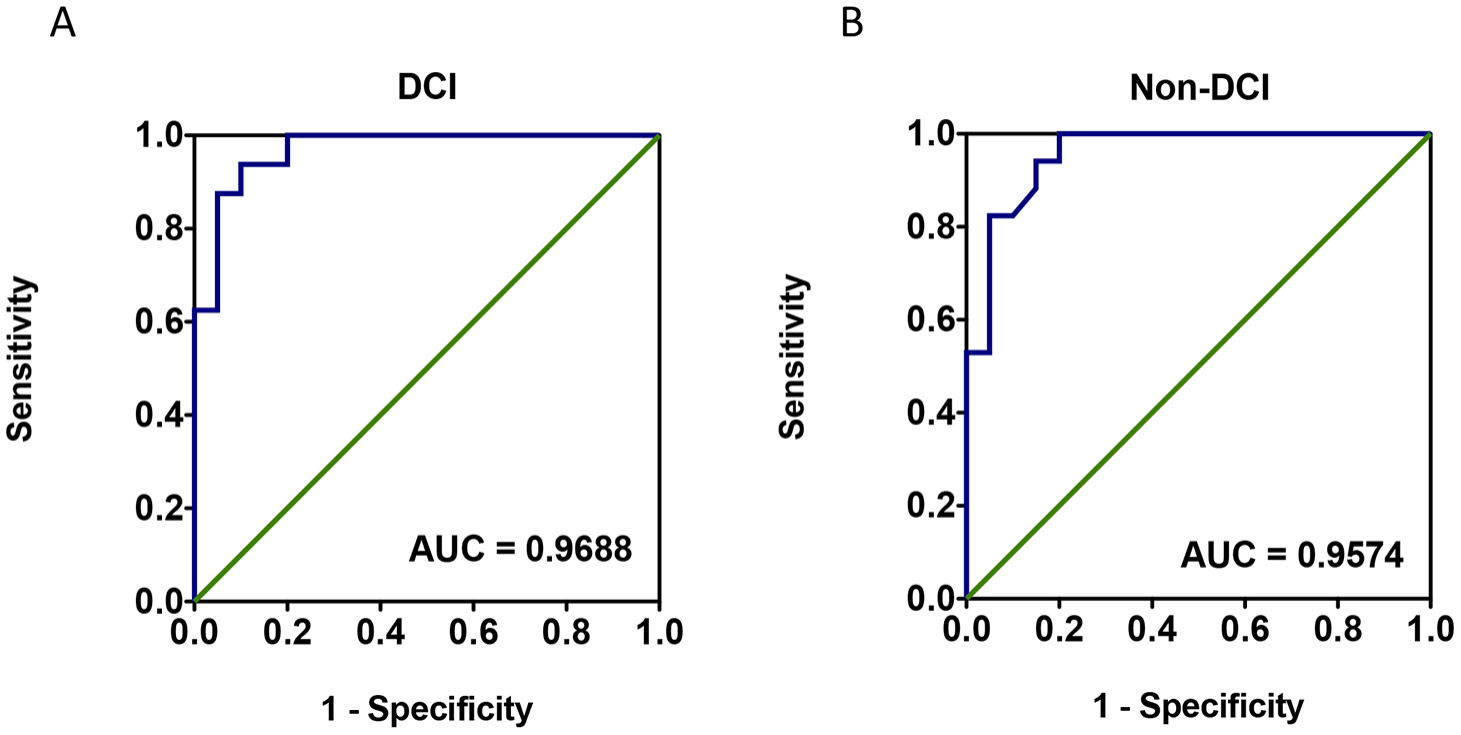
Receiver operating characteristic (ROC) curve of miR-324-3p for aneurysmal subarachnoid hemorrhage patients with delayed cerebral infarction (A) and without delayed cerebral infarction (B) using raw ΔCt values. *p < 0.05. AUC = Area under the curve.

To assess the circulating miRNA-132 and miRNA-324 in SAH and healthy subjects, qPCRs were performed with individual samples from the same subject cohorts (N = 20 for SAH DCI group, N = 20 for SAH non-DCI group, and N = 20 for healthy control group) (Tables 1 and 2). In order to eliminate the possible influence from the selected housekeeping gene (internal control) U6, C. Elegans miRNA cel-miR-39 (Takara, Japan) was added into the samples as a spike-in external control. It was shown that miR-132 presented a 9.5 fold (95%CI: 2.3 to 16.7) upregulation in SAH DCI group and 3.4 fold (95%CI: 1.0 to 5.8) upregulation in Non-DCI group (Fig 2). For miR-324, it presented a 4924 fold (95%CI: 2620 to 7228) upregulation in SAH DCI group and a 4545 fold (95%CI: 2408 to 6683) upregulation in Non-DCI group (Fig 3). The trends of change in these two miRNAs were found to be consistent with what was shown in the microarray (GEO accession number GSE72340 and S1 File). From the ROC curve of miR-324, the area under the curve (AUC) was 0.97 for DCI group and 0.96 for Non-DCI group (Fig 4). miR-132 gave an AUC of 0.75 for DCI group and 0.73 for non-DCI group (Fig 5). However, there were no significant differences in fold changes between SAH patients with and without DCI [fold change ratios (mean+/-SD): 2.7+/-4.2 and 1.1+/-1.1 for miRNA-132 and miRNA-324].

## Discussions

### Differential miRNA expression in SAH patients reflects the result of hemorrhage, but not the etiology

We demonstrated that 99 miRNAs were found to be dysregulated in the SAH patient group with CI using the Affymetrix miRNA 3.0 array. 81 miRNAs were upregulated and 18 were downregulated (GEO accession number GSE72340 and S1 File). Findings from the KEGG pathway analysis showed that miRNAs and target genes for axon guidance and TGF-beta signaling were involved (S2 File). Individual genes from these pathways, including Netrin-1 and TGF-beta-1 were found to have increased expressions after neurological damage after stroke. Netrin-1 involves in neural repair and angiogenesis regulation [33], while TGF-beta-1 was demonstrated to be another powerful angiogenesis regulator [34]. These identified pathways also aligned with the fact that SAH patients might develop delayed cerebral infarction, which involved ischemic injury. Our microarray data was obtained from two pooled serum samples from the healthy control group (N = 20) and SAH patients DCI group (N = 20). This initial study provided qualitative data to guide the second part of the study.

### Differential expression of miR-132-3p and miR-324-3p in DCI group and non-DCI group SAH patients

miR-132 involves in angiogenesis in several *in vitro* and animal studies [35][36][37], which downregulates the expression of p120RasGAP, a negative regulator of Ras, thereby suppressing endothelial cell proliferation and vessel growth [36]. This miRNA is also a brain-enriched miRNA in humans and is involved in a number of neurological conditions [38][39][40][41]. miR-132 leads to neuronal apoptosis in Alzheimer’s Disease when downregulated [42]. We showed that as compared to healthy control, miR-132-3p is 9.5-fold upregulated (p < 0.05) in SAH DCI group and 3.4-fold upregulated in SAH non-DCI group. miR-324-3p was found to be upregulated in a cerebral ischemia study using MCAO (Middle Cerebral Artery Occlusion) Rat model [43][44]. In the qPCR study, miR-324-3p was undetectable in 15 of the healthy control individuals, which aligned with the finding from microarray. Imputation of a Ct value of 40 was performed on the 15 samples with undetectable signals according to a recent publication [45] for downstream statistical analysis. Our interpretation was supported by two previous studies that miR-324-3p was at low expression levels in blood samples of healthy controls in two other previous studies [46] [47]. We suggested that miR-324-3p might be a potential biomarker for SAH (AUC of ROC = 0.97 for DCI group and 0.96 for Non-DCI group), though not for DCI. Future investigations should be performed to confirm the uniformity with a different cohort using standard curves for absolute qPCR quantification.

### Limitations

Firstly, the control was recruited from the family members of SAH patients with the no major medical problem, and without smoking and hypertension history. During the recruitment, we could not know which miRNAs would turn out to be the candidates and thus were not matched for selected known possible modifiying factors. In two miRNA expression profile studies[48] [49], both miRNA 324-3p and miRNA 132-3p were not different between smoker and control. There were 2 smokers in the SAH with delayed cerebral infarction group and no smokers in the SAH without delayed cerebral infarction group. Removing these two smokers from analysis did not alter the study results significantly. In the miRNA expression profile study by Li et al. [50], hypertension was related to a 0.59 fold change decrease in miRNA 324-3p. The inclusion of hypertension patients thus will not confound the increase associated with SAH. The miRNA fold change was slightly higher in non-hypertensive SAH patients than hypertensive SAH patients (p = 0.665), which was compatible with the results of Li et al. [50]. Regression analyses was not performed due to limitation in sample size. Secondly, Day 7 was selected for blood withdrawal as it was conceived to be the most relevant time point to investigate the pathological process related to DCI. It remains possible that other miRNAs may be actively involved in earlier phase. Also, the exact timing of evoluation of DCI is variable and day 7 may not capture the exactly the same phase of development among different patients. An earlier blood sample for miRNAs may be useful to further elucidate the roles of miRNA 324-3p and miRNA 132-3p. Thirdly, although there were differences in fold changes in miRNA 324-3p and miRNA 132-3p between DCI and non-DCI groups, they were not statistically significant. Forthly, due to limitation in sample size, hypertension, smoking, Fisher grade, intraventricular hemorrhage, hydrocephalus, and size of aneurysm were not included as a factor for analysis. The control and SAH patients were also not matched with the current method of recruitments.

### Conclusion

Our study demonstrated that as compared to control, miR-132 presented a 9.5 fold upregulation in SAH DCI group and 3.4 fold upregulation in Non-DCI group. miR-324 presented a 5458 fold upregulation in SAH DCI group and a 4545 fold upregulation in Non-DCI group. However, the differences between the SAH DCI and non-DCI groups were not statistically significant.

## Supporting Information

S1 File List of 88 dysregulated miRNA comparing SAH patient DCI group and healthy control group of microarray data from Affymetrix miRNA 3.0 array. (DOCX)

S2 File List of circulating miRNA with dysregulated expression levels and their target genes after bioinformatics analysis with DIANA-miRPath v.2.0. (DOCX)
