# Supplementary material for: Circulating microRNA 132-3p and 324-3p Profiles in Patients after Acute Aneurysmal Subarachnoid Hemorrhage
Source: PLoS One. 2015 Dec 16;10(12):e0144724. doi: 10.1371/journal.pone.0144724 (PMC4682983; doi:10.1371/journal.pone.0144724)
Supplement: S1 File — (DOCX) [file pone.0144724.s001.docx]

**Supplementary file**

S.1 List of 99 dysregulated miRNA comparing aneurysmal subarachnoid hemorrhage patients with delayed cerebral infarction group and healthy control group of microarray data from Affymetrix miRNA 3.0 array.

| 1.5-FOLD OR MORE UPREGULATED |  |
| --- | --- |
| Probe Set ID | **Fold change Δ(DCI group-Control group)** |
| hsa-miR-222_st | **40.723** |
| hsa-miR-15a_st | **24.068** |
| hsa-miR-1268_st | **22.225** |
| hsa-miR-574-5p_st | **18.175** |
| hsa-miR-27b_st | **15.152** |
| hsa-miR-4463_st | **14.136** |
| hsa-miR-125a-5p_st | **13.565** |
| hsa-miR-4454_st | **11.760** |
| hsa-miR-502-3p_st | **10.654** |
| hsa-miR-152_st | **10.560** |
| hsa-miR-4532_st | **9.573** |
| hsa-miR-99b_st | **9.572** |
| hsa-miR-27a_st | **9.003** |
| hsa-miR-182_st | **8.791** |
| hsa-miR-4651_st | **7.406** |
| hsa-miR-181b_st | **7.257** |
| hsa-miR-199b-3p_st | **7.010** |
| hsa-miR-30e_st | **6.094** |
| hsa-miR-143_st | **5.917** |
| hsa-miR-4433_st | **5.829** |
| hsa-miR-199a-3p_st | **5.765** |
| hsa-miR-500a-star_st | **5.713** |
| hsa-miR-223_st | **5.442** |
| hsa-miR-145_st | **5.075** |
| hsa-miR-3178_st | **4.936** |
| hsa-miR-339-3p_st | **4.709** |
| hsa-miR-320e_st | **4.679** |
| hsa-miR-660_st | **4.354** |
| hsa-miR-29a_st | **4.173** |
| hsa-miR-324-5p_st | **4.019** |
| hsa-miR-4443_st | **3.438** |
| hsa-miR-629_st | **3.398** |
| hsa-miR-194_st | **3.346** |
| hsa-miR-4429_st | **3.337** |
| hsa-miR-4741_st | **3.320** |
| hsa-miR-532-5p_st | **3.155** |
| hsa-miR-409-3p_st | **3.095** |
| hsa-miR-4763-3p_st | **3.044** |
| hsa-miR-30d_st | **2.879** |
| hsa-miR-4745-5p_st | **2.748** |
| hsa-let-7g_st | **2.613** |
| hsa-miR-451_st | **2.583** |
| hsa-miR-762_st | **2.540** |
| hsa-miR-30c_st | **2.524** |
| hsa-miR-574-3p_st | **2.406** |
| hsa-miR-28-3p_st | **2.403** |
| hsa-miR-1290_st | **2.353** |
| hsa-miR-501-3p_st | **2.344** |
| hsa-miR-132_st | **2.309** |
| hsa-miR-30b_st | **2.307** |
| hsa-miR-3656_st | **2.273** |
| hsa-miR-510_st | **2.262** |
| hsa-miR-16_st | **2.253** |
| hsa-miR-342-3p_st | **2.210** |
| hsa-miR-18b_st | **2.207** |
| hsa-miR-210_st | **2.170** |
| hsa-miR-106b_st | **2.144** |
| hsa-miR-1275_st | **2.090** |
| hsa-miR-425-star_st | **2.041** |
| hsa-miR-4484_st | **1.989** |
| hsa-miR-4281_st | **1.973** |
| hsa-miR-1469_st | **1.952** |
| hsa-miR-342-5p_st | **1.916** |
| hsa-miR-4706_st | **1.899** |
| hsa-miR-4488_st | **1.827** |
| hsa-miR-4270_st | **1.816** |
| hsa-miR-28-5p_st | **1.802** |
| hsa-miR-21_st | **1.795** |
| hsa-miR-1228-star_st | **1.776** |
| hsa-miR-486-5p_st | **1.762** |
| hsa-miR-4306_st | **1.728** |
| hsa-miR-92b_st | **1.618** |
| hsa-miR-20b_st | **1.612** |
| hsa-miR-199a-5p_st | **1.604** |
| hsa-let-7i_st | **1.599** |
| hsa-miR-149-star_st | **1.593** |
| hsa-miR-4508_st | **1.576** |
| hsa-miR-1301_st | **1.567** |
| hsa-miR-378c_st | **1.561** |
| hsa-miR-345_st | **1.532** |
| hsa-miR-4695-5p_st | **1.509** |
|  |  |
| 1.5 fold OR MORE DOWNREGULATED |  |
| hsa-let-7d_st | **-1.567** |
| hsa-miR-744_st | **-1.630** |
| hsa-miR-151-5p_st | **-1.731** |
| hsa-miR-150_st | **-1.745** |
| hsa-let-7a_st | **-2.142** |
| hsa-miR-1280_st | **-2.404** |
| hsa-miR-3162-3p_st | **-2.564** |
| hsa-miR-3613-3p_st | **-2.713** |
| hsa-miR-4530_st | **-2.723** |
| hsa-miR-940_st | **-2.746** |
| hsa-miR-378h_st | **-3.211** |
| hsa-miR-1281_st | **-3.233** |
| hsa-miR-4668-5p_st | **-3.432** |
| hsa-miR-4487_st | **-4.578** |
| hsa-miR-3613-5p_st | **-6.535** |
| hsa-miR-4455_st | **-7.660** |
| hsa-miR-4668-3p_st | **-8.709** |
| hsa-miR-553_st | **-10.409** |
