# Supplementary material for: Circulating microRNA 132-3p and 324-3p Profiles in Patients after Acute Aneurysmal Subarachnoid Hemorrhage
Source: PLoS One. 2015 Dec 16;10(12):e0144724. doi: 10.1371/journal.pone.0144724 (PMC4682983; doi:10.1371/journal.pone.0144724)
Supplement: S2 File — (DOCX) [file pone.0144724.s002.docx]

**Supplementary file**

| S.2 LS.2 List of circulating miRNA with dysregulated expression levels and their target genes after bioinformatics analysis with DIANA-miRPath v.2.0. | | | |  |
| --- | --- | --- | --- | --- |
| **KEGG pathway (ID)** | **Regulated miRNAs** | **Target Genes** | |  |
| ***Prion disease (hsa05020)*** | miR-152, miR-4436b-5p, miR-4775 | EGR1, NCAM2, PRNP, LAMC1, C9, MAPK1, PRKACB | |  |
|  |  |  | |  |
| ***ErbB signaling pathway (hsa04012)*** | miR-155-5p, miR-4454, miR-152, miR-199b-3p, miR-199a-3p, miR-4429, miR-409-3p, miR-4492, miR-330-3p, miR-1290, miR-4465-5p, miR-421, miR-1301, miR-339-5p, miR-4449, miR-4530, miR-940, miR-4487, miR-4775, miR-4668-3p | CAMK2D, BRAF, GSK3B, HBEGF, PRKCA, ERBB2, SOS2, STAT5A, CAMK2G, NRAS, CRKL, NRG4, CRK, SHC1, PIK3CB, PAK2, TGFA, PIK3R5, PAK7, RAF1, CDKN1B, EIF4EBP1, EGFR, KRAS, ARAF, RPS6KB2, CAMK2A, NCK1, PTK2, CBLB, PIK3R3, NCK2, MAPK8, AKT1, NRG3, SOS1, PIK3CG, SRC, PAK4, PAK6, BTC, NRG1, PRKCB, GAB1, SHC4, AKT3, CAMK2B, PIK3CA, MAP2K1, MTOR, MAP2K4, MAPK1, ABL2, ERBB4, MAPK10, RPS6KB1, EREG | |  |
| ***Axon guidance (hsa04360)*** | miR-155-5p, miR-502-3p, miR-4689, miR-4739, miR-4741, miR-532-5p, miR-4429, miR-4492, miR-762, miR-330-3p, miR-501-3p, miR-342-5p, miR-199a-5p, | EFNB2, SEMA6A, PLXNA2, GSK3B, ABLIM3, ROCK1, RAC2, NRAS, PAK2, NGEF, ARHGEF12, EPHA8, SEMA5A, EPHA5, ROCK2, PAK7, ROBO2, NTN1, SEMA3C, SEMA4F, SRGAP1, EFNA3, PLXNA1, LRRC4C, PPP3CC, RHOA, KRAS, FYN, EFNA5, EPHA7,ROBO3, SEMA3F, NFATC4, SEMA4G, NCK1, PPP3CA, PTK2, RASA1, UNC5A, EFNB3, NFAT5, SLIT2, DCC, SRGAP3, EPHA3, DPYSL5, DPYSL2, NFATC2, UNC5C, CFL2, SEMA3D, NRP1, RAC1, PAK4, CDC42, PAK6, SEMA4B, UNC5B, SEMA3A, EFNB1, EPHB6, PLXNC1, SEMA7A, EPHA6, LIMK1, ABLIM1, SEMA4D, SEMA6D, RND1, SLIT3, EPHA4, NFATC3, SEMA3E, UNC5D, MAPK1, NFATC1, EPHB1, GNAI1, PPP3R2, EFNA1 | |  |
|  | miR-494, miR-28-5p, miR-1280, miR-940, miR-4472, miR-4307, miR-4775, miR-4668-3p |  |  |  |
|  |  |  |  |  |
|  |  |  |  |  |
|  |  |  |  |  |
|  |  |  |  |  |
|  |  |  |  |  |
| ***TGF-beta signaling pathway*** | miR-145-5p, miR-21-5p, miR-4793-3p, miR-152, miR-139-5p, miR-409-3p, miR-330-3p, miR-342-3p, | TGFBR1, ID2, ROCK1, SMAD2, INHBB, SMAD9, THBS1, THBS2, PPP2CA, SMURF2, BMPR1B, BMP5, ROCK2, PITX2, SMAD3, CUL1, INHBA, CDKN2B, ID4, BMP6, RHOA, ACVR1, SKP1, RPS6KB2, DCN, SMAD4, E2F5, RBL1, SMAD5, ACVR2A, GDF6, TFDP1, SP1, ACVR1C, TGFB2, EP300, PPP2CB, BMPR1A, THBS3, SMAD7, NOG, BMP7, MAPK1, PPP2R1B, LEFTY2, TGFBR2, BMPR2, RPS6KB1 | |  |
| ***(hsa04350)*** | miR-494, miR-4734, miR-4449, miR-940, miR-4307, miR-4668-5p, miR-4775, miR-4668-3p |  |  |  |
|  |  |  |  |  |
|  |  |  |  |  |
|  |  |  |  |  |
| ***Neurotrophin signaling pathway (hsa04722)*** | miR-125b-5p, miR-21-5p, miR-155-5p, miR-4440, miR-125a-5p, miR-199b-3p, miR-199a-3p, miR-320e, miR-4429, miR-409-3p, miR-330-3p, miR-1290, miR-28-5p, miR-339-5p, miR-4530, miR-940, miR-4668-5p, miR-4307, miR-3613-5p, miR-4775, miR-4668-3p | CAMK2D, BRAF, GSK3B, NTRK2, NTF3, SOS2, RIPK2, SH2B3, CAMK2G, CAMK4, NRAS, CRKL, YWHAE, IRAK3, CALM3, CRK, SHC1, PIK3CB, MAPK7, MAP2K7, SORT1, RAP1A, BAX, NTRK3, FRS2, RAF1, BCL2, RPS6KA1, MAP3K1, RHOA, MAGED1, KRAS, BDNF, CALM2, FASLG, RPS6KA5, CAMK2A, PSEN2, JUN, PIK3R3, TP73, MAPK8, TRAF6, AKT1, KIDINS220, NTRK1, ARHGDIA, RPS6KA6, PIK3R1, SOS1, PIK3CG, PTPN11, IRS1, RAC1, CDC42, PSEN1, RPS6KA3, GAB1, SHC4, MAPK12, AKT3, CAMK2B, PIK3CA, FOXO3, MAP2K1, PRDM4, RELA, IRAK1, MAPK1, RAP1B, MAP3K5, MAPK10 | |  |
|  |  |  |  |  |
|  |  |  |  |  |
|  |  |  |  |  |
| ***Dopaminergic synapse***  ***(hsa04728)*** | miR-502-3p, miR-199b-3p, miR-4739, miR-199a-3p, miR-4429, miR-409-3p, miR-4745-5p, miR-1290, miR-501-3p, miR-1275, miR-4668-3p, miR-553 | FOS, GSK3B, PRKCA, GNG13, PPP2R5E, KIF5A, GNGT1, PPP2R3A, PPP2R2C, PPP2CA, CREB5, PPP1CC, GRIA1, PPP2R5D, PPP3CC, DDC, PPP2R2D, PPP2R5A, GRIA2, CAMK2A, PPP3CA, ARNTL, PLCB1, MAPK8, AKT1, PPP2R2A, SCN1A, PPP2R1A, PRKACA, ITPR1, PPP2R3C, CACNA1C, DRD5, GSK3A, PRKCB, GNAL, GNAQ, AKT3, CAMK2B, CREB3L2, GRIA4, CREB3L1, GNG4, GRIN2A, PLCB4, CLOCK, KIF5B, GNAI1, PPP1CB, GRIA3 | |  |
|  |  |  |  |  |
|  |  |  |  |  |
|  |  |  |  |  |
|  |  |  |  |  |
|  |  |  |  |  |
|  |  |  | |  |
| **Listed target genes are experimentally validated in published literatures.* | | |  | |
| **Analysis with DIANA-miRPathv.2.0* | |  | |  |
